# Modeling and quality assessment of nystagmus eye movements recorded using an eye-tracker

**DOI:** 10.3758/s13428-020-01346-y

**Published:** 2020-04-20

**Authors:** William Rosengren, Marcus Nyström, Björn Hammar, Markus Rahne, Linnea Sjödahl, Martin Stridh

**Affiliations:** 1 Department of Biomedical Engineering, Lund University, Lund, Sweden; 2 Lund University, Humanities Lab, Lund, Sweden; 3 Department of Ophtalmology, Lund University, Lund, Sweden; 4 Faculty of Engineering, Lund University, Lund, Sweden

**Keywords:** Nystagmus, Modelling, Eye tracking

## Abstract

Mathematical modeling of nystagmus oscillations is a technique with applications in diagnostics, treatment evaluation, and acuity testing. Modeling is a powerful tool for the analysis of nystagmus oscillations but quality assessment of the input data is needed in order to avoid misinterpretation of the modeling results. In this work, we propose a signal quality metric for nystagmus waveforms, the *normalized segment error* (NSE). The NSE is based on the energy in the error signal between the observed oscillations and a reconstruction from a harmonic sinusoidal model called the *normalized waveform model* (NWM). A threshold for discrimination between nystagmus oscillations and disturbances is estimated using simulated signals and receiver operator characteristics (ROC). The ROC is optimized to find noisy segments and abrupt waveform and frequency changes in the simulated data that disturb the modeling. The discrimination threshold, *𝜖*, obtained from the ROC analysis, is applied to real recordings of nystagmus data in order to determine whether a segment is of high quality or not. The NWM parameters from both the simulated dataset and the nystagmus recordings are analyzed for the two classes suggested by the threshold. The optimized *𝜖* yielded a true-positive rate and a false-positive rate of 0.97 and 0.07, respectively, for the simulated data. The results from the NWM parameter analysis show that they are consistent with the known values of the simulated signals, and that the method estimates similar model parameters when performing analysis of repeated recordings from one subject.

## Introduction

Nystagmus is a symptom expressed as involuntary oscillating eye movements with a reported prevalence of 24 per 10,000 people in the general population (Sarvananthan et al., 2009). The nystagmus symptoms may lead to decreased visual acuity and in certain cases to *oscillopsia*, which is a sensation that the world is in motion (Leigh & Zee, 2015). For some of those who are affected, the symptoms are persistent. Traditionally, nystagmus is divided into acquired nystagmus, which develops later in life due to for example trauma, and early onset nystagmus, which is developed before or a few months after birth (Hussain, 2016; McLean et al., 2007).

Diagnostics of nystagmus is difficult and often requires long clinical experience. In order to evaluate the condition of a person with nystagmus, various methods, both automatic and manual, for diagnostics and evaluation of the nystagmus signal patterns have been developed (Leigh & Zee, 2015; Dell’Osso & Daroff, 1975; Dell’Osso & Jacobs, 2002). These methods may involve classification of the eye movement dynamics, often referred to as the *waveform*. The waveform may provide insights into the underlying cause of the nystagmus symptoms (Leigh & Zee, 2015). In this work, the waveform, as measured by an *eye-tracking* system, is considered on a cycle-to-cycle basis.

Eye tracking is a technology used to estimate the gaze direction or to measure eye movements (Holmqvist et al., 2011). The technology has previously been used in nystagmus research, and various metrics for evaluation of nystagmus, based on eye-tracking data, have been constructed. Many of these methods utilize automatic processing of the recorded eye-movement signals. Automatic processing of nystagmus signals is an established and alternative analysis method compared to manual inspection of nystagmus oscillations. The analysis techniques originate from a wide range of different disciplines such as control theory (Broomhead et al., 2000), dynamic systems modeling (Akman et al., 2005), time series analysis (Theodorou & Clement, 2016) and time-frequency analysis (Hosokawa et al., 2004). One may consider two different approaches to model nystagmus: *system-based* and *signal-based* modeling. System-based modeling is aimed at the mechanisms of the nystagmus itself. Such methods are often concerned with modeling of, e.g., the saccadic system, and investigates possible mechanisms behind nystagmus (Broomhead et al., 2000; Akman et al., 2005; Abadi et al., 1997; Harris & Berry, 2006). Signal-based modeling of nystagmus data is aimed at modeling and classification of measured nystagmus oscillations, i.e., waveforms. Such models may be used for classification of recorded signals into different established waveform morphologies (Theodorou & Clement, 2016; Abadi & Worfolk, 1991), in order to determine the visual function of a person with nystagmus (Dell’Osso & Jacobs, 2002; Felius et al., 2011), or to evaluate the effect of a treatment (Young & Huang, 2001). Manual classification of cycle-to-cycle waveform morphologies have previously been investigated (Dell’Osso & Daroff, 1975), where 12 different waveform morphologies observed in people with nystagmus are described.

**Fig. 1:**
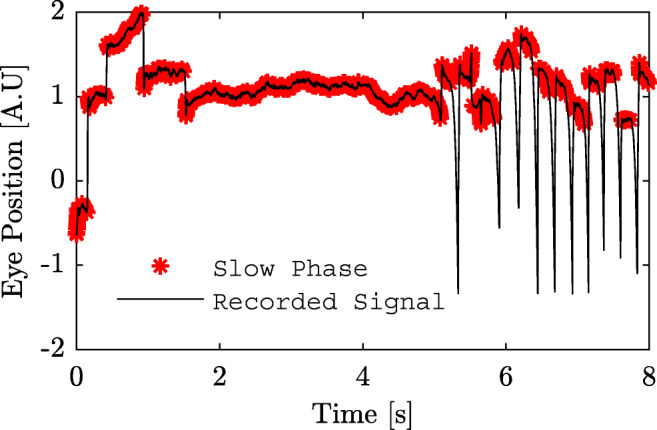
Example of slow phase detection. The slow phase detection method from Rosengren et al., (2019) applied to a nystagmus signal. The *red asterisks* show the segments of detected slow phases. As can be seen, the segment without an oscillatory pattern in the recorded signal is erroneously labeled as a slow phase

When modeling nystagmus eye movements, it is desirable to only include segments in the data that contribute to the overall understanding of the underlying condition. Therefore, an *exclusion criterion* for the data may be introduced. In this context, an exclusion criterion refers to the method for which data in a study is excluded from further analysis. In this work, exclusion of data is considered on a sub-signal level, meaning that some segments in a single recording may be included whereas other segments may be excluded from further analysis. In order to illustrate the importance of data exclusion, consider the eye movement recording in Fig. 1. Here, a slow phase detector (Rosengren et al., 2019) based on velocity leads to detection of slow phases in the non-oscillatory part of this data segment, due to the low velocity of this part. Even though a low velocity is an indicator or a slow phase, it is undesirable to use this segment to describe the nystagmus oscillations of this specific person, or to use this segment for calibration. It is therefore desirable to have a method that automatically rejects segments in the recorded signals that do not exhibit an oscillating pattern and thus should not be included in the analysis.

Removing segments of non-oscillating data from recordings is a process that has previously been used in nystagmus research. A method for removal of blinks using position, velocity and acceleration thresholds has been implemented for calibration purposes (Dunn, 2014). The threshold is based on the standard deviation of the recorded signals.

In order for this method to be operative, at least one blink needs to be present in the recording (Dunn, 2014). A threshold for the nystagmus cycle length has been used as an inclusion criterion on a signal segment level (Theodorou and Clement, 2016). The cycle length was identified using a method using periodic orbits, where the main cycle length was determined as the peak in a histogram of the periodic orbits. All cycle lengths within ± 12.5 ms of the main cycle length were included in the analysis.

Although some methods exist for detection of segments in nystagmus eye-movement recordings that should be excluded from further analysis, no systematic investigation with the specific purpose to evaluate the performance of the data exclusion methods has to our knowledge been conducted. This means that there is very little information on how well these methods work for excluding undesirable data segments. Data exclusion is an important component when characterizing nystagmus oscillations, since the reliability of automatic analysis methods may be significantly influenced by how the method handles disturbances and ’non-nystagmus’ segments. If the exclusion criterion allows too many segments to enter further analysis, there is a risk of modeling segments that do not represent the nystagmus oscillation of the patient. On the other hand, if the exclusion criterion allows too few segments, there is a risk of not having enough data left for subsequent analysis, as well as of missing dynamic changes, such as waveform and frequency changes, in the recording.

In this work, we present a signal-based method for modeling of nystagmus signals with the purpose to select segments for further analysis. In order to do so, a metric called the *normalized segment error* (NSE) is introduced. The modeling method is based on a harmonic sinusoidal model, and the NSE is computed as the normalized error between the reconstructed signal and the recorded eye-movement signal. An evaluation dataset consisting of simulated signals is created and used for performance evaluation. The method is also evaluated on a dataset with recordings from participants with nystagmus.

This paper is organized as follows: In Section “Proposed model”, the model of the nystagmus oscillations is presented. The datasets used for evaluation of the proposed model are described in detail in Section “Datasets”. The evaluation strategy for the proposed method is presented in Section “Model performance evaluation”. Finally, the results are presented and the method is discussed in Sections “Results” and “Discussion”, respectively.

## Proposed model

The model considered in this work is based on a pseudo-stationary assumption of the nystagmus signal. Consider the harmonic sinusoidal model *s*[*n*] with *H* harmonics (Stridh et al., 2009),

1 $$ s[n] = {\sum}_{h=1}^{H} s_{h}[n], $$

where

2 $$ s_{h}[n] = a_{h}\sin[2 \pi (f_{1} h) n + \phi_{h}] $$

and, *a*_*h*_ is the amplitude of the *h*:th harmonic, *f*_1_ is the first harmonic frequency and *ϕ*_*h*_ is the phase of harmonic *h*, and *n* = 0,…,*N* − 1.

The estimation of the model parameters consists of two steps. The first step describes the data preprocessing and the extraction of harmonic components, see Section “Preprocessing”. The second step, the parameter estimation, is described in Section “Block model parameter estimations”. A description of the model features used for analysis of the nystagmus recordings is presented in Section “Waveform features”. Finally, the NSE is defined in Section “The normalized segment error (NSE)”.

### Preprocessing

The preprocessing stage consists of two parts. First, the signal is downsampled to 100 Hz (original sampling rate is 1000 Hz for the data presented in this work). The second step is to high-pass filter the signal using a third-order Butterworth filter with a cutoff frequency of 2 Hz, removing frequencies lower than the cutoff. The preprocessed signal is denoted *s*_*p*_[*n*].

In order to estimate the different harmonic components, *s*_*h*_[*n*], each component needs to be extracted from the preprocessed signal. After the preprocessing, the global first harmonic frequency of the signal, $\hat {F}_{1}$, is estimated using Welch spectrum estimation with an overlap of 50 % and a segment length of 512 samples (for a 100-Hz signal). The harmonic components, *s*_*h*_[*n*], are computed as *s*_*p*_[*n*] filtered through a Kaiser bandpass filter, *B*_*h*_(*f*,*F*) with the following design settings:

3 $$ B_{1}(f, F)=\left\{\begin{array}{ll} 1, & \text{if } \big|F - f \big| \leq f_{w1}\\ 0, & \text{if } \big|F - f \big| > f_{w2} \end{array}\right. $$

for the first harmonic, and

4 $$ B_{h}(f, F)=\left\{\begin{array}{ll} 1, & \text{if } Fh - (1 + \delta_{h}) \leq f \leq Fh + (1+ \delta_{h})\\ 0, & \text{if } Fh - (2 + \delta_{h}) \leq f \leq Fh + (2 + \delta_{h}) \end{array}\right. $$

for *h* > 1, where $\delta _{h} = \frac {(h-1)} 2$. In this work, *f*_*w*1_ = 1.3 and *f*_*w*2_ = 2.3 (Stridh et al., 2009).

### Block model parameter estimations

As stated above, the frequency of the nystagmus signal is in general not stationary. The signal is therefore divided into short segments of length *N*_*b*_. The choice of the segment length is considered with the following tradeoff: if the segment length is too short, it may result in poor parameter estimates, and if it is too long, the stationarity assumption of each segment may not be valid. The latter problem is addressed by using a method of overlapping segments, while reconstructing the signal for a shorter interval. The segment length was set to *N*_*b*_ = 67 (corresponding to 0.67 s).

Another issue is that the frequency estimate $\hat {F}_{1}$ is not necessarily representative for all intervals in the recorded signal. If the first harmonic frequency varies more than ± 1.3 Hz, the output energy of the affected segments may be severely reduced. In order to remedy this, two additional sets of harmonic components are computed for the frequencies $\hat {F}_{0} = \hat {F}_{1} - 2.6$ and $\hat {F}_{2} = \hat {F}_{1} + 2.6$. The frequency estimate for the time interval ***n***_*b*_ = [*n*_0_,…,*n*_0_ + *N*_*b*_ − 1] is determined by maximizing the first harmonic energy,

5 $$ \hat{F}[\boldsymbol{n}_{b}] = \arg\max_{\hat{F}_{i}} \bigg[E(\hat{F}_{0})[\boldsymbol{n}_{b}], E(\hat{F}_{1})[\boldsymbol{n}_{b}], E(\hat{F}_{2})[\boldsymbol{n}_{b}]\bigg], $$

where

6 $$ E\big(\hat{F}_{i}[\boldsymbol{n}_{b}]\big) = {\sum}_{k=n_{0}}^{n_{0} + N_{b} -1}\bigg| s_{1}^{(i)}[k] \bigg|^{2} $$

and $s_{1}^{(i)}[\boldsymbol {n}_{b} ]$ is the resulting first harmonic signal after *s*_*p*_[***n***_*b*_] is filtered through *B*_1_(*f*,*F*), and where $F = \hat {F}_{i}$. The signal, however, is reconstructed for a time interval *n*_*c*_ ∈ [*l* − *c*_0_,*l* + *c*_0_] where the overlap *c*_0_ is computed as

7 $$ c_{0} = \frac {N_{b}} {2\hat{F}[\boldsymbol{n}_{b}]}, $$

*n*_0_ is the start sample for the interval of length *N*_*b*_ and

8 $$ l = n_{0} + \frac {N_{b}} 2. $$

This means that every approximate wave is reconstructed separately based on a window around it. The frequency, *f*_*h*_, and phase, *ϕ*_*h*_, of each harmonic are estimated according to Stridh et al., (2009)

9 $$ \hat{f}_{h} = \arg\max_{f}\bigg|{\sum}_{n_{b}=n_{0}}^{n_{0} + N} s_{h}[n_{b}]e^{-j 2 \pi f n_{b}} \bigg| $$

and

10 $$ \hat{\phi}_{h} = \text{arctan}\bigg(- \frac{{\sum}_{n_{b}=n_{0}}^{n_{0} + N}s_{h}[n_{b}]\sin(2 \pi \hat{f}_{h} n_{b})} {{\sum}_{n_{b}=n_{0}}^{n_{0} + N}s_{h}[n_{b}]\text{cos}(2 \pi \hat{f}_{h} n_{b})}\bigg) $$

The amplitude, $\hat {a}_{h}$, for the *h*:th harmonic is estimated from the analytic signal transformation (Sörnmo and Laguna, 2005),

11 $$ \hat{a}_{h} = \frac 1 {2 c_{0}} {\sum}_{i=l - c_{0}}^{l + c_{0}-1} |\tilde{s}_{h}[i]| $$

where $\tilde {s}_{h}[i]$ is the analytical transformation of *s*_*h*_.

The signal is not stationary unless $\hat {f}_{h} = h \hat {f}_{1}, \forall h$, which is generally not going to be the case. In order to create a stationary model, Eq. 1 is rewritten as

12 $$ s[n_{b}] = {\sum}_{h=1}^{H} a_{h}\sin[2 \pi (\hat{f}_{1} h) n_{b} + 2 \pi (\hat{f}_{h} - \hat{f}_{1} h)n_{b} + \hat{\phi}_{h}] $$

where $\hat {f}_{h}$ is the frequency estimate of harmonic *h*. The second argument of the sinusoid, $2 \pi (\hat {f}_{h} - \hat {f}_{1} h) n_{b}$, may be viewed as a phase component. In order for this to be stationary, the index *n*_*b*_ is replaced by a fixed index value, for example the block center index *l* (Stridh et al., 2009). This results in the model

13 $$ s^{\prime}[n_{b}] = {\sum}_{h=1}^{H} \hat{a}_{h}\sin[2 \pi (\hat{f}_{1} h) n_{b} + \hat{\phi}_{h}^{\prime}] $$

where

14 $$ \phi_{h}^{\prime} = 2 \pi (\hat{f}_{h} - \hat{f}_{1} h)l + \hat{\phi}_{h}. $$

### Waveform features

When the model parameters have been estimated, it is possible to reconstruct and compare waveforms with different frequencies, amplitudes, or morphologies. The relationship between the amplitude coefficients in a harmonic model is a direct measure of the influence of each respective harmonic. For example, if the first harmonic amplitude is much greater compared to the amplitudes of the other harmonics, the resulting signal will be close to a pure sinusoid. Two waveforms may be similar in morphology, but where the absolute amplitudes are quite different. In order to compare waveform morphologies of a harmonic model, all amplitudes are normalized according to

15 $$ \hat{R}_{h} = \frac {\hat{a}_{h}} {\hat{a}_{1}}. $$

The first harmonic phase is a measure of where the oscillation in a given segment begins. However, when comparing different waveform morphologies, it is not of interest to study the absolute phase, but rather to study the relative phase values. In order to compare different waveform morphologies, the phases of all harmonics are adjusted to the first harmonic phase according to Stridh et al., (2009):

16 $$ \hat{\phi}_{h}^{\prime\prime} = \frac {\hat{\phi}_{h}^{\prime}} h. $$

Once all phases have been rescaled to the first harmonic, the angular difference, $\delta _{\phi _{h}^{\prime \prime }}$, between the first harmonic phase and rescaled harmonic phases are computed according to Stridh et al., (2009):

17 $$ \hat{\delta}_{\phi_{h}^{\prime\prime}} = \hat{\phi}_{h}^{\prime\prime} - \hat{\phi}_{1}^{\prime\prime} = \hat{\phi}_{h}^{\prime\prime} - \hat{\phi}_{1}^{\prime} $$

A new model, the *normalized waveform model* (NWM), may be written as

18 $$ s_{a}[n_{b}] = {\sum}_{h=1}^{H} \hat{R}_{h} \sin(2 \pi \hat{f}_{1}hn_{b} + \hat{\delta}_{\phi_{h}^{\prime\prime}}h) $$

### The normalized segment error (NSE)

As described in Section “Introduction”, the NSE is introduced in order to determine whether a signal segment should be considered for further analysis. The NSE of a segment with length *N*_*s*_ is computed as

19 $$ NSE_{s} = \frac {{\sum}_{n_{s}=n_{0}}^{n_{0} + N_{s}-1} |s^{\prime}_{p}[n_{s}] - s^{\prime}[n_{s}]|^{2}} {{\sum}_{n_{s}=n_{0}}^{n_{0} + N_{s}-1} |s^{\prime}_{p}[n_{s}]|^{2}} $$

where

20 $$ s^{\prime}_{p}[m] = s_{p}[m] - \frac 1 {N_{s}} {\sum}_{n_{s}=n_{0}}^{n_{0} + N_{s}-1} s_{p}[n_{s}] $$

and

21 $$ s^{\prime}[m] = s[m] - \frac 1 {N_{s}} {\sum}_{n_{s}=n_{0}}^{n_{0} + N_{s}-1} s[n_{s}]. $$

The signals *s*_*p*_[*n*] and *s*[*n*] denote the preprocessed and reconstructed signals, respectively, and *N*_*s*_ is the segment length. If *NSE*_*s*_ > *𝜖* for some value *𝜖*, the segment should be excluded from further analysis. The choice of the *𝜖* value is further discussed in Section “ROC analysis”.

## Datasets

In this section, the two datasets used for the evaluation of the proposed method are presented. In Section “Evaluation dataset”, the evaluation dataset (ED), which consists of simulated signals, is described in detail. The purpose of this dataset is to evaluate the proposed method on signals with known characteristics and known disturbances, which is important both for evaluation of the parameter estimation process and to set the threshold of the exclusion criterion. The content of the recorded participant dataset, PD, is presented in Section “Participant dataset”.

### Evaluation dataset

In order to evaluate the performance of the proposed method, it is tested on a dataset with known reference signals. The analysis software for the evaluation dataset was written in Python (version 2.7) using the SciPy (version 1.0.0) signal processing library.

In order to use signals that resemble real nystagmus waveforms, the illustrations of nystagmus waveforms presented in Dell’Osso and Daroff (1975) were digitized using image processing. The digitized signals were then parametrized using Fourier analysis in order to introduce signal modulations as well as to be able to track model parameter values. The different waveforms were captured as images using a print screen function on a 27-inch (5120 x 2880) resolution screen and transformed into one-dimensional signals. A detailed description of the parametrization of the different waveforms is found in Appendix together with the phase and amplitude values for each waveform type.

In order to study the performance of the modeling method, simulated template signals were created and corrupted by various signal modulations. Three different types of modulations were introduced: amplitude modulation, frequency shift, and noise consisting of white Gaussian noise.

**Fig. 2:**
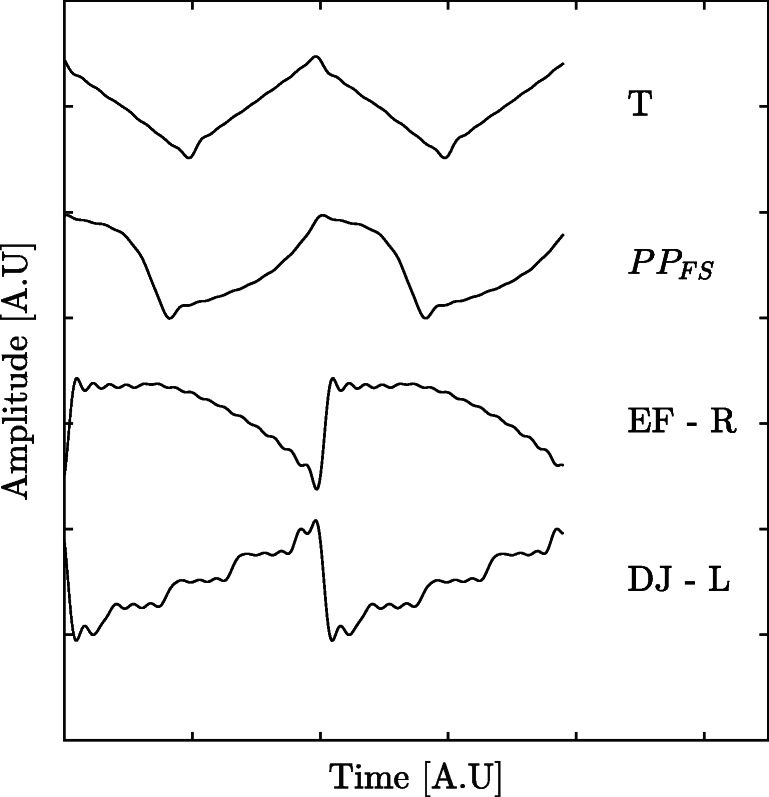
Evaluation dataset waveforms. The four different waveforms used to create the evaluation dataset were dual jerk - left (DJ-L), extended foveation - right (EF-R), pseudo pendular with foveating saccades (PP_FS_) and triangular (T). All waveforms have been reconstructed using Fourier analysis

A total of 50 test signals, each consisting of the four template signals DJ-L, EF-R, PP_FS_ and T (Dell’Osso & Daroff, 1975), with a total duration for each test signal of 24 s, were generated. The template waveforms were chosen as the signal parameters are reasonably different from each other, and it was desirable to test the method for different types of waveforms. Examples of these four waveforms are found in Fig. 2. Each test signal comprised of four *waveform components*, where each waveform component consisted of one of the four waveforms DJ-L, EF-R, PP_FS_ or T. The duration of each waveform component was 6 s and the sampling frequency was set to 1000 Hz. The order of the different waveform component was randomized for each 24-s signal (four waveform components × 6 s). The initial frequency of each of waveform component was set to 6 Hz. The model, *y*[*n*], for the amplitude modulation and frequency shift is given by

22 $$ y[n] = A[n]{\sum}_{h=1}^{H} a_{h}\sin[2 \pi (f_{1}[n] h) n + \phi_{h}], $$

where *A*[*n*] is the amplitude variation and *f*_1_[*n*] is the time-dependent frequency for each waveform component.

23 $$ A[n] = \sin[2 \pi f_{A} n + \text{cos}(2 \pi \frac{f_{A}} {5} n )] $$

24 $$ f_{1}[n] = \left\{\begin{array}{ll} 6,& \text{if } n \leq n_{1}\\ f[u],& \text{if } n_{1} < n \leq N, \end{array}\right. $$

For each waveform component, there was one frequency shift, occurring between 1.5 s and 4.5 s after the onset of the waveform component. The new frequency, f[u], was randomly sampled from a uniform distribution, $\mathcal {U} \big (8, 10 \big )$.

The number of noise episodes in each simulated signal was randomized where the maximum number of occurrences was six times for every waveform component. The distribution of the noise was a zero mean white noise Gaussian distribution, $\mathcal {N} \big (0, 0.05\big )$. The time of occurrence for each noise episode was also randomized, and it was possible for multiple episodes to occur simultaneously. The onset and offset times for the frequency shifts and noise episodes were stored for all waveform components. The variables for the simulated signals are presented in Table 1.

**Table 1:** The random variable values for the evaluation dataset

*f*[*u*]	*n*_1_	*f*_*A*_	*w*_*a*_	*d*_*w*_
$\mathcal {U} \bigg (8, 10 \bigg )$	$\mathbb {Z} \in [1500, 4500]$	$ \mathcal {U}\bigg (10^{-2}, 10^{-1}\bigg )$	$\mathcal {N} \bigg (0, 0.05\bigg )$	$\mathbb {Z} \in [500, 1000]$

**Fig. 3:**
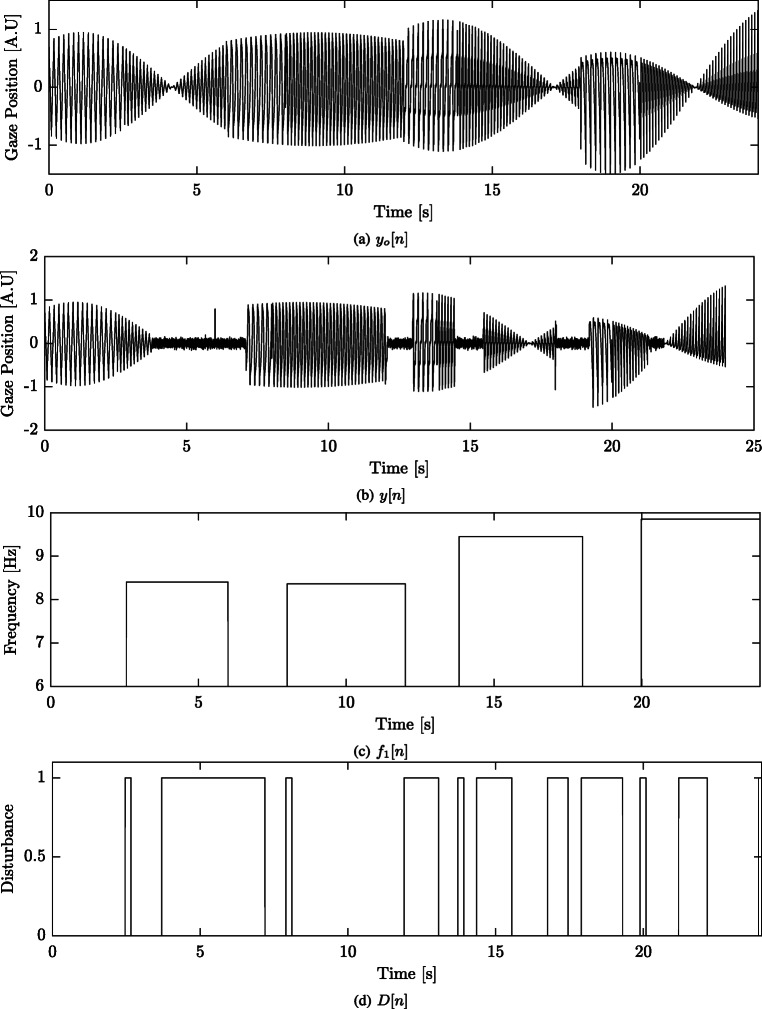
Illustration of the simulated signals. **a** The original simulated signal *y*_*o*_[*n*], **b** the ’observed’ simulated signal *y*[*n*], **c** the frequency *f*_1_[*n*] and the **d** disturbance vector *D*[*n*] are illustrated

A *disturbance vector*, *D*[*n*] is defined as

25 $$ D[n] = \left\{\begin{array}{ll} 1, & \text{if } t_{on} - \delta_{t} \leq n \leq t_{off} + \delta_{t}\\ 0, & \text{if } otherwise \end{array}\right. $$

where *t*_*on*_ and *t*_*off*_ are the onset and offset times of either a frequency modulation or a noise segment. The term *δ*_*t*_ is an additional disturbance time added due to the moving window of the modeling method. For this work, *δ*_*t*_ = 100 samples. An example of a simulated signal is presented in Fig. 3. The original signal without any disturbances, *y*_*o*_[*n*], is plotted in Fig. 3a. The change in frequency, waveform, and the noise segments are illustrated in Fig. 3b. The corresponding disturbance vector, *D*[*n*], is plotted in Fig. 3d.

### Participant dataset

The dataset containing nystagmus recordings, PD, consists of two sub-datasets denoted PD_1_ and PD_2_. In short, these datasets were recorded with the following setup. Uncalibrated pupil and corneal reflection data were recorded binocularly at 1000 Hz using an EyeLink 1000 Plus eye tracker in desktop mode, with the host software v. 5.09 and the DevKit v. 1.11.571. The eyes were tracked using the center of mass mode. The geometry of the experiment setup was in accordance with the manufacturer’s recommendations (SR-Research, 2010). The stimuli software was written in Python and PsychoPy (version 1.83) (Peirce, 2007). An ASUS VG248QE monitor with a resolution of 1920 × 1080 pixels, with dimensions 53 × 30 cm was used for stimuli presentation and all participants were seated 80 cm from the screen. The head was stabilized using a chin and forehead rest. The study is approved by the ethics board at Lund University and all experiments are in accordance with the Declaration of Helsinki.

All participants were subject to calibration and validation before the experiment started. During the calibration, the participants were instructed to focus on a set of nine calibration targets, appearing in a randomized order. The vertical target positions were ± 10^∘^ and 0^∘^ and the horizontal target positions were ± 18^∘^ and 0^∘^. The same procedure was implemented during the validation using four different targets appearing in random order. Both the horizontal and vertical positions of the validation targets were ± 5^∘^. During both the calibration and the validation, each eye was first recorded monocularly (the other eye was covered) followed by a binocular recording. A black circle with radius 0.6^∘^ with a red circle of radius 0.15^∘^ in the center were used as the calibration target. The color of the background was gray.

During the experiment, the participants were presented with fixation targets in five different positions: (− 16^∘^, 0^∘^), (16^∘^, 0^∘^), (0^∘^, 0^∘^), (0^∘^, − 8^∘^) or (0^∘^, 8^∘^), where the first coordinate is the horizontal position and the second is the vertical position and the coordinate (0^∘^, 0^∘^) is at the center of the screen. The same target composition (black circles with red centers) as for the calibration was used and each target was shown for 15 s, except for the center target which was shown for 30 s. After each target had been shown, a target at the central position, (0^∘^, 0^∘^), was shown for 5 s. The analysis of these segments has not been included in this work. In between each target, the participant was allowed to rest the eyes and blink for 5 s. This dataset is referred to as PD_1_. In total, recordings from five male participants with diagnosed early onset nystagmus were included (*M* = 34.9 [years], *SD* = 14.7 [year]). The participants were diagnosed by a senior neuro-ophthalmologist [BH] at Skåne University Hospital, Lund, Sweden.

A second dataset, PD_2_, was created by performing repeated experiments for one of the participants from PD_1_ (eight times on different days). The data from one session were excluded due to equipment malfunction. The same calibration, validation, and fixation recording procedure as were used for PD_1_ were also used for PD_2_, where the horizontal calibration positions were ± 16^∘^ and 0^∘^. The age of this male participant was 25 years. PD_2_ was created in order to investigate the repeatability of the estimated model parameters and thereby the measured nystagmus pattern. All participants were calibrated using the foveation detection and the Procrustes calibration method presented in Rosengren et al., (2019).

## Model performance evaluation

The performance evaluation is divided into two different parts. The first part, presented in Section “Evaluation dataset analysis”, is the analysis of the ED. In Section “Participant dataset analysis” examples of the modeling performance for participant dataset PD_1_ and PD_2_ are presented. All signals are analyzed using the NSE. The *𝜖* estimated from the ED, see Section “Evaluation dataset analysis”, is used in the analysis of the PD_1_ and PD_2_. The NSE is computed in 200-ms segments, which corresponds to half of a cycle of an oscillation at 2.5 Hz. Segments in the data for which the EyeLink system cannot track the pupil are marked by the system. At each such occurrence, 200 ms before and after the occurrence were removed from the signal. This occurs for example during a blink. All segments for which the time between two episodes of missing data was less than 5 s were excluded from analysis.

### Evaluation dataset analysis

#### ROC analysis

As described in Section “Evaluation dataset”, three different signal modulations have been introduced in the ED. Two of these, the frequency shift and the noise, lead to segments in the dataset that should be excluded from further analysis. Just before and after an abrupt change in frequency, there is no ’pure’ waveform, which is why these segments in the signals should be excluded from further analysis.

The goal of the modeling is to find these episodes in the recorded signal by utilizing the NSE of each reconstructed signal segment. In order to use the NSE as an inclusion criteria, an NSE threshold that maximizes the number of segments that contains nystagmus waveforms (true-positive rate) and at the same time minimizes the number of segments with the unwanted waveform modulations (false-positive rate) is constructed. For this purpose, the *receiver operating characteristics* (ROC) is used as follows:

26 $$ \mathcal{C} = \left\{\begin{array}{ll} 1, & \text{if } NSE_{s} > \epsilon\\ 2, & \text{if } NSE_{s} \leq \epsilon \end{array}\right. $$

A true positive (TP) is defined if *D*[*n*_0_] = … = *D*[*n*_0_ + *N*_*d*_ − 1] = 1 and $\mathcal {C} = 1$ in Eq. 26. A false positive (FP) is defined if $\mathcal {C} = 1$, where *D*[*n*_0_] = … = *D*[*n*_0_ + *N*_*d*_ − 1] = 0. A true negative (TN) is defined if $\mathcal {C} = 2$ and *D*[*n*_0_] = … = *D*[*n*_0_ + *N*_*d*_ − 1] = 0 whereas a false negative (FN) is defined if $\mathcal {C} = 2$ and *D*[*n*_0_] = … = *D*[*n*_0_ + *N*_*d*_ − 1] = 1. In this work, the length of each disturbance segment, *N*_*d*_, equals 20 samples (200 ms).

The true-positive rate (TPR) is defined as

27 $$ \text{TPR} = \frac{\text{TP}} {\text{TP} + \text{FN}} $$

and the false-positive rate (FPR) is defined as

28 $$ \text{FPR} = \frac{\text{FP}} {\text{FP} + \text{TN}} $$

All segments that contain both undesired episodes (noise, frequency, or waveform change) and desired episodes (nystagmus oscillations) were excluded from the analysis. The optimal *𝜖* was determined by

29 $$ \hat{\epsilon} = \arg\min_{d_{\epsilon}}\bigg(d_{\epsilon}\bigg) $$

where

30 $$ d_{\epsilon} = \sqrt{(1 - TPR(\epsilon))^{2} + FPR(\epsilon)^{2}}. $$

#### Frequency analysis

The frequency estimation is a crucial part of the model parameter estimation. If the frequency estimation is poor, there is a risk that the bandpass filters (see Section “Preprocessing”) use the wrong passband, which in turn may lead to poor estimation of the other model parameters. The bandpass filters have a theoretical binary gain of 1 if the frequency is within the passband and 0 if the frequency is outside. This means that if

31 $$ |\hat{f}_{1} - f_{w1}| \leq 1.3, $$

the spectral energy is large. The filtering process is evaluated by determining the percentage of segments for each class of the two classes $\mathcal {C}=1$ and $\mathcal {C}=2$, where the gain in Eq. 3 is equal to 1.

#### Amplitude analysis

The distributions of the amplitude ratios $\hat {R}_{2}$ and $\hat {R}_{3}$ are estimated using *kernel density estimation* (KDE) and are compared to the true values of *R*_1_ and *R*_2_. The KDEs are estimated using a Gaussian kernel with a bandwidth 0.05. If the modeling is accurate, it is expected that the energy of the distribution for the accepted values are centered around the true parameter values. At the same time, it is expected that there is a larger variance of the estimated values for the rejected segments.

### Participant dataset analysis

There are three properties of the proposed model that are desired to study using the participant datasets. The first property is the ability to model different waveforms from different individuals. The second property is the ability to replicate results from one individual over multiple recordings. The last property is the ability to differentiate waveforms recorded from different spatial positions, i.e., fixation targets. The PD_1_ is used for evaluation of the first property whereas the PD_2_ is used for evaluation of all three properties.

In order to study these properties, the parameters of the normalized waveform model are analyzed. Contrary to the analysis performed for the ED, there are no reference parameters to compare with for these two datasets. Instead of comparing the parameter values to known reference values, the distributions of the estimated model parameters are studied. The parameters $\hat {R}_{2}$ and $\hat {\delta }{\phi }^{\prime \prime }_{2}$ for each recording are used to compute a polar coordinate pair according to:

32 $$ \begin{array}{@{}rcl@{}} x &=& \hat{R}_{2} \text{cos}(\hat{\delta}{\phi}^{\prime\prime}_{2}),\\ y &=& \hat{R}_{2} \sin (\hat{\delta}{\phi}^{\prime\prime}_{2}). \end{array} $$

Using the *𝜖* estimated in Section “ROC analysis”, each polar coordinate pair is classified as either accepted or rejected.

**Fig. 4:**
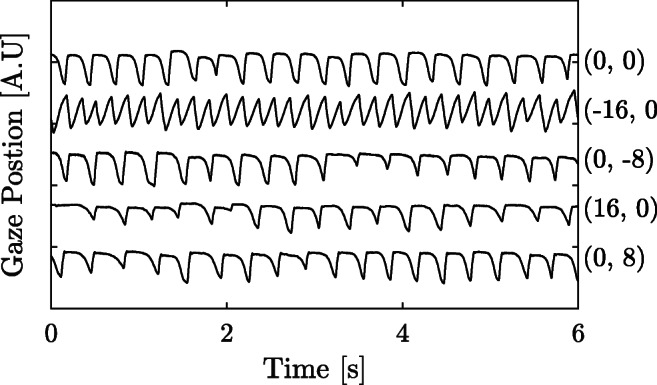
Example signals from PD_2_. The signals represent recordings from five different fixation positions (for one participant). The coordinate pair after each signal describes at which fixation target position the signals were recorded. The frequency and waveform of the signal recorded at position (− 16^∘^,0^∘^) is different compared to the other four

In order to study the spatial performance of the normalized waveform model, a *representative waveform* from each fixation target recording is reconstructed from the model parameters. An example of the variance in spatial waveforms from PD_2_ is illustrated in Fig. 4. The representative waveform is reconstructed using the densities of the $\hat {f}_{1}$, $\hat {R}_{2}$, $\hat {R}_{3}$, $\hat {\delta }{\phi }^{\prime \prime }_{2}$ and $\hat {\delta }{\phi }^{\prime \prime }_{3}$ parameters. For the first three parameters, KDEs are estimated from all estimated parameter values from the recording of one fixation target. The parameter values maximizing each respective KDE are used for the representative waveform. The KDEs were estimated using a Gaussian kernel with a bandwidth of 0.5 for the estimation of $\hat {f}_{1}$ and a bandwidth 0.05 for the estimation of the amplitudes ratios. For the angular parameters, $\hat {\delta }{\phi }^{\prime \prime }_{2}$ and $\hat {\delta }{\phi }^{\prime \prime }_{3}$, a circular histogram was used instead. The bandwidth of the circular histogram was set to 5^∘^.

## Results

The Results section is divided into two parts, where the ED results are presented in Section “Evaluation dataset” and results for PD_1_ and PD_2_ are presented in Section “Participant data”.

### Evaluation dataset

The results for the ED are organized in the following structure: First, the choice of *𝜖*, determined by Eq. 29, is presented. Based on this *𝜖*, the performance of the frequency and amplitude ratio estimations are analyzed.

#### Choice of *𝜖*

**Fig. 5:**
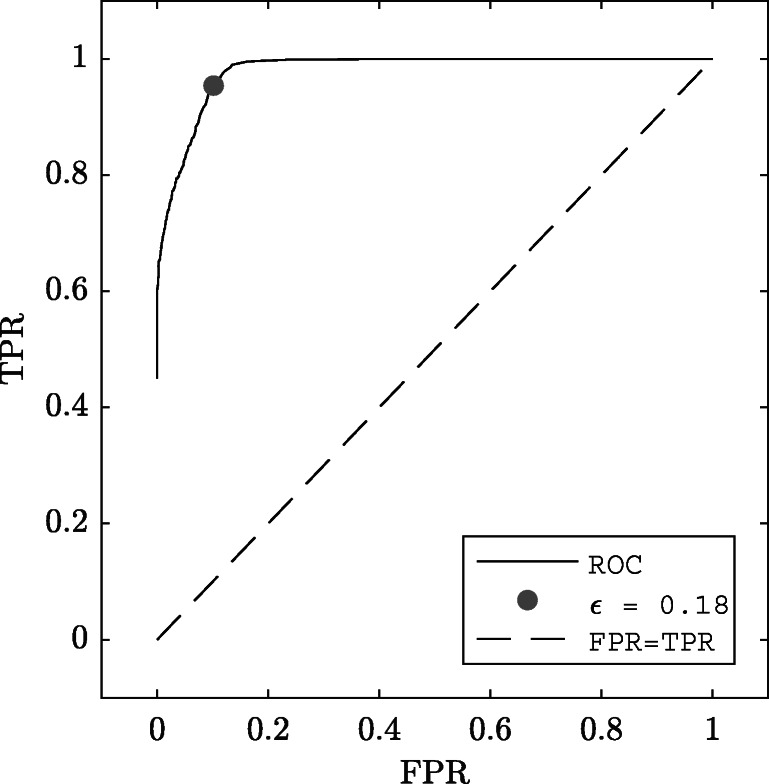
Receiver operating characteristics. The false-positive rate (FPR) is plotted against the true-positive rate (TRP) for various values of the error threshold. The position on the ROC minimizing equation (30) is plotted as a *gray circle*

The ROC analysis of *𝜖* is presented in Fig. 5. Out of the blocks that were considered for the ROC analysis, the prevalence of corrupted episodes (excluding amplitude modulation) was 44 %. The total number of analyzed segments was 4295.

The *𝜖* that minimizes the distance *d* in Eq. 30 is marked with a circle. The corresponding TPR and FPR values for *𝜖* = 0.18 are TPR = 0.97 and FPR = 0.07. The interpretation of this is that 97 % of all segments of noise, frequency, or waveform changes are detected and that 7 % of all segments where a non-oscillating signal was detected, are true nystagmus oscillations. This *𝜖* is used for all subsequent analysis, both for the ED and the PD datasets. All segments where *𝜖* > 0.18 are labeled as rejected ($\mathcal {C}=1$), and all other segments are label as accepted ($\mathcal {C}=2$).

#### Frequency and amplitude ratio estimation performance

The results show that out of the accepted segments, 99.7 % of the blocks satisfy the criterion in Eq. 31. Only 65 % of the rejected segments satisfy this criterion. This means that 99.7 % of the accepted segments are within the allowed bandwidth of the bandpass filters.

**Fig. 6:**
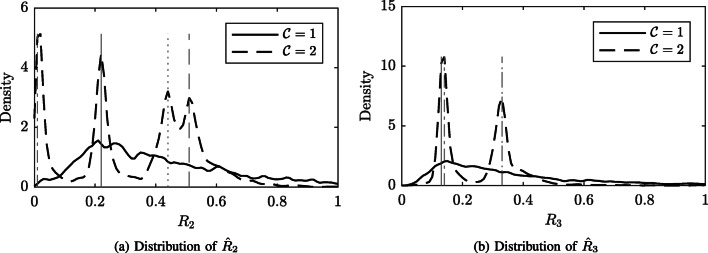
KDE of $\hat {R}_{2}$ and $\hat {R}_{3}$ for the ED. The estimated distributions of the amplitude ratios $\hat {R}_{2}$ and $\hat {R}_{3}$ are presented in Figs. 6a, 6b for the two classes rejected segments ($\mathcal {C}=1$) and accepted segments ($\mathcal {C}=2$). The *vertical gray lines* represent the known reference values for each of the four waveforms. Note that the *R*_3_ value for the DJ-L and EF-R are almost identical

The distributions of the estimated $\hat {R}_{2}$ and $\hat {R}_{3}$ are plotted in Fig. 6. The distribution of $\hat {R}_{2}$ and $\hat {R}_{3}$ for accepted segments ($\mathcal {C}=2$, dashed line) matches the known references values well. The distribution of the estimated $\hat {R}_{2}$ and $\hat {R}_{3}$ for rejected segments ($\mathcal {C}=1$, full line) has a larger variance compared to the accepted data. The peaks at the positions of the known values are not clearly distinguishable for the rejected segments.

### Participant data

The percentage of rejected segments for the participant datasets PD_1_ and PD_2_ are presented in Table 2. As can be seen, the average percentage of rejected segments are 47 % and 29 % for PD_1_ and PD_2_, respectively. For one participant in PD_1_, 90 % of the total number of segments for the five fixation target recordings were rejected. The average rejection rate of PD_1_ (47 %) data is similar to the average rejection rate of the ED data (44 %).

**Table 2:** The proportion of rejected segments for ED, PD_1_ and PD_2_

Dataset	Average	Lowest	Highest
ED	0.44	0.27	0.58
PD_1_	0.47	0.19	0.90
PD_2_	0.29	0.22	0.41

The lowest proportion of rejected segments is quite similar for the two datasets PD_1_ and PD_2_. The highest proportion of rejected segments, however, is quite different 0.9 and 0.41, respectively. The results are presented on a participant level, meaning that the total proportion of the rejected segments for one participant (five fixation target recordings for each participant) is presented for each of the datasets

**Fig. 7:**
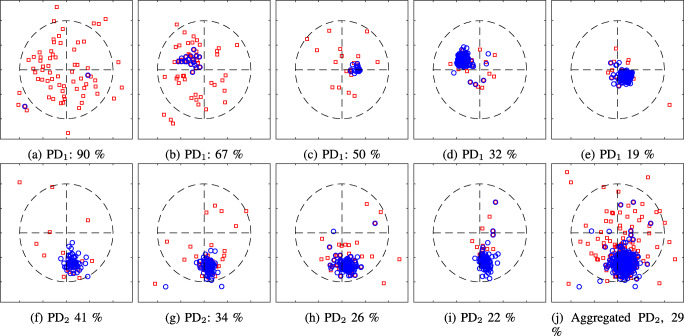
Polar coordinates from signals with varying rejection rates and waveform morphologies. The plots above show the estimated $\hat {R}_{2}$ and $\hat {\delta }^{\prime \prime }_{\phi _{2}}$ plotted as polar coordinates for five recordings from PD_1_ (*top row*) and four recordings and the aggregated estimations (Figure (j) from PD_2_ (*bottom row*). The percentage in each caption shows the overall exclusion rate for each participant recording. The parameters have been estimated from a recording of the primary position (0^∘^, 0^∘^). The *blue circles* represent the accepted segments and the *red squares* represent the rejected segments

The polar representations of $\hat {R}_{2}$ and $\hat {\delta }{\phi }^{\prime \prime }_{2}$ are plotted in Fig. 7, where the top and bottom rows show example recordings from PD_1_ and PD_2_, respectively. The rejected segment parameter estimations are plotted as red squares and the accepted segments parameters are plotted as blue circles. Two general trends can be observed in these figures. First, high rejection rates lead to a larger spread of values within the unit circle. For example, all estimated values in Fig. 7e (19 % rejection rate) are more concentrated than the parameter values presented in Fig. 7a (90 % rejection rate). This means that the waveform morphology for the data presented in Fig. 7e varies to a lower degree compared to that in Fig. 7a. Note that Fig. 7j illustrates the aggregated parameter estimations for all recordings in PD_2_. Second, the repeatability of the two parameter values $\hat {R}_{2}$ and $\hat {\delta }{\phi }^{\prime \prime }_{2}$ appears to be high for high-quality signals. All of the included parameter values from the PD_2_ dataset (the bottom row) are positioned at approximately the same location inside the unit circle. There is one important thing to note: the $\hat {R}_{2}$ values have been limited to $\hat {R}_{2}\leq \sqrt {2}$ in these plots. In some cases, the $\hat {R}_{2}$ value is much larger than this restriction. If this is the case, then the segment is most often classified as rejected ($\mathcal {C}=1$). These results illustrate the individual variation in waveform morphology during one measurement and the reproducibility properties of the model between repeated measurements.

**Fig. 8:**
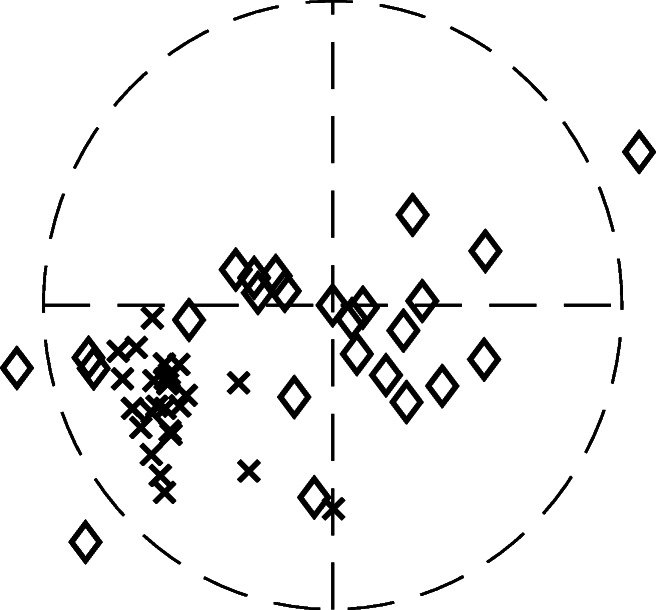
Illustration of polar coordinates. The $\hat {R}_{2}$ and $\hat {\delta }_{\phi ^{\prime \prime }_{h}}$ values from the signal in Fig. 1 are plotted as the radius and angle, respectively. The parameters estimated from the first 5 s of the signal are plotted as *crosses*, whereas the rest of the parameters are plotted as *diamonds*

The polar coordinate representation of $\hat {R}_{2}$ and $\hat {\delta }{\phi }^{\prime \prime }_{2}$ of the signal shown in Fig. 1, is presented in Fig. 8. The diamonds represent the oscillations after 5 s whereas the crosses represent the oscillations up until 5 s. As illustrated in Fig. 8, there is a relatively large variance of the parameter values for the crosses compared to the diamonds. All diamonds have similar radius and angle, whereas the crosses are spread out over the entire unit circle. This indicates that when there is an oscillatory pattern with a repetitive waveform in the data, the model parameters are clustered together. However, when there is no such pattern, the model parameters diverge. These results may be used to determine whether there is a stable oscillating pattern in the data or not.

**Fig. 9:**
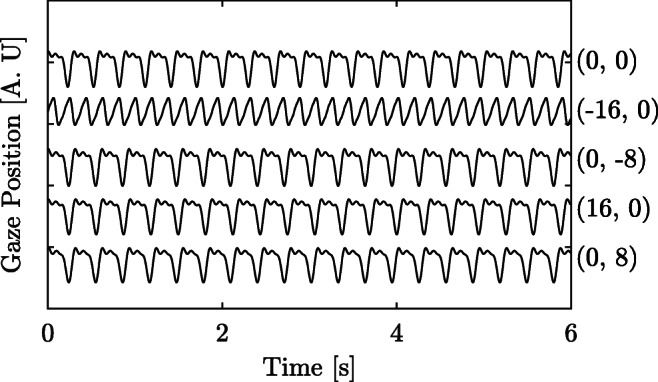
Example reconstruction of the participant dataset (PD_2_) waveforms. The reconstruction of the representative waveforms in Fig. 4 for each of the five spatial positions. The *x*-axis represents time and the *y*-axis represents the position of the fixation targets where the data were recorded

The reconstruction of the waveforms for the PD_2_ data is illustrated in Fig. 9. As is shown in Fig. 4 (the original signals), the eye movement recorded at position (− 16^∘^,0^∘^) is significantly different compared to the other four fixation recordings. This is true for all the repeated recordings in PD_2_. The change in nystagmus characteristics on the left side of the screen for this patient has been captured in the signal reconstruction (Fig. 9). This implies that the normalized waveform model is useful for analysis of and comparison between different nystagmus waveforms. These results are an illustration of the spatial properties of the model.

**Fig. 10:**
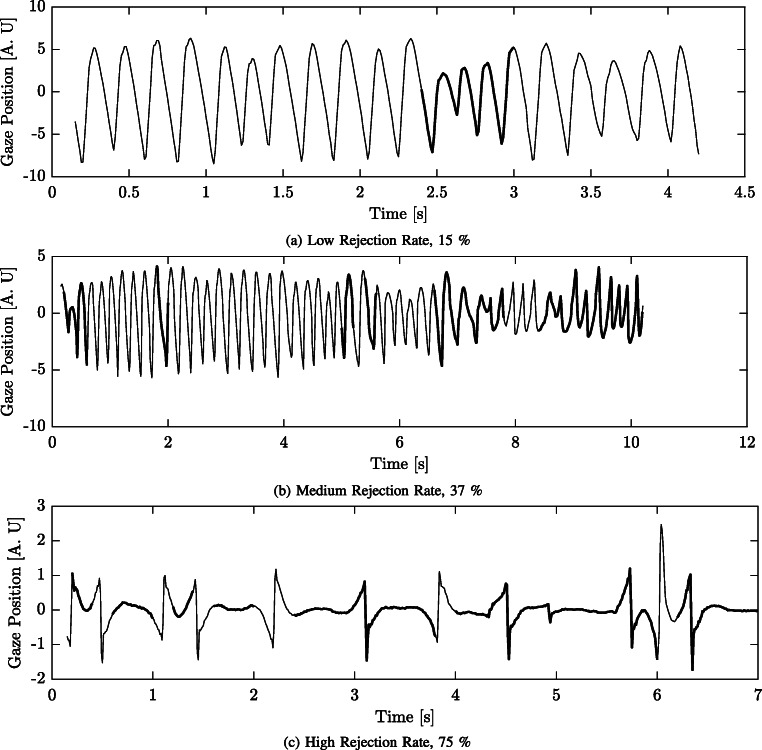
Examples of different rejection rates. Three different levels of rejection rates are presented in Figs. 10a–c. The rejected segments are plotted as *thick lines*. Note that the length of the different signals varies. The title states the rate of rejection for each signal

In Fig. 10, examples of rejected cycles from the PD_1_ and PD_2_, are presented. The thick full line intervals in the signals represent segments that are rejected from further analysis. Figure 10a illustrates that segments that are too non-stationary are rejected from analysis. The same type of pattern is found in Fig. 10b. In Fig. 10c, most of the signal is rejected. This is likely due to a too low frequency of the oscillation that is highly affected by the high-pass filter used in the preprocessing stage.

## Discussion

In this work, we have presented a method for assessment of signal quality, referred to as the NSE, for use in eye-tracking recordings from nystagmus patients. The NSE is used to exclude data segments that are undesirable to model due to for example recording artifacts. The nystagmus oscillations are modeled using a harmonic sinusoidal model. The signal is divided into segments and the NSE is assessed for each segment where a high NSE value suggests that the segment should be excluded from further analysis. The method is validated by analysis of simulated signals, which have been synthesized from previously reported waveform templates (Dell’Osso and Daroff, 1975). Various frequency and amplitude modulations as well as abrupt waveform changes and noise have been introduced and the presented method is evaluated based on its ability to identify these segments. The accuracy of the frequency and amplitude estimations has been evaluated for the simulated signals. Finally, model parameter estimation has been evaluated on recordings from nystagmus patients.

There are four aspects of the method presented in this work that are of significance for the modeling of nystagmus oscillations. First, the NSE allows automatic detection of segments that should be rejected from further analysis. As illustrated in Fig. 6 and by the ROC computations, the NSE is able to capture segments that represent the underlying nystagmus oscillation, while rejecting segments that do not contain an oscillatory signal. Note that the signal quality and NSE discussed in this work are different compared to the *waveform quality* presented in Dell’Osso and Jacobs (2002). In that work, waveform quality is related to the results obtained from a visual acuity test for different nystagmus waveforms.

Second, the NSE allows the users of this method to compare different segments to each other, and use the segments that are of ‘hi gh quality’, .i.e., that has a low NSE. This metric may be used to rate different segments in terms of their quality, and focus the analysis to the segments with the highest quality. Third, the harmonic model has proved itself to be useful when reconstructing waveforms. As can be seen in Fig. 9, the presented model is able to capture differences in the waveforms of the signals observed in Fig. 4. These results suggest that the model is appropriate for capturing various nystagmus waveforms, which means that the method could be used as a clinical diagnostic tool.

Finally, the method does not need calibrated data in order to work. This is a great advantage, since calibration of nystagmus patients is often hard (Rosengren et al., 2019), and the results of calibration are often unreliable. The method may also be used as a preprocessing step in order to find segments with a high signal quality to be used for calibration.

The frequency estimation results that are presented in Section “Frequency and amplitude ratio estimation performance” suggest that inaccurate frequency estimation for segments with high NSE is 35 %, and at the same time, that almost all (99.7 %) of the segments with a low NSE provide accurate frequency estimates. This is important since the method relies on accurate frequency estimations in order to work properly. The filtering method presented in Eq. 6 was developed specifically for nystagmus signals. In PD_1_ and PD_2_, the frequency range of most oscillations were between 3 and 6 Hz. It is, however, possible to find nystagmus oscillations with frequencies outside this range (Leigh & Zee, 2015). A possible alternative to the filter bank approach presented in this work was presented in Buttu et al., (2013). This method was developed for electrocardiogram (ECG) analysis and would likely need to be adjusted for nystagmus analysis.

The results of the ROC analysis suggest that the NSE assessment works well to separate oscillatory segments from corrupt segments. In this work, the optimum ROC value was estimated by computing the distance to the top left corner of the ROC coordinate system. The optimization of *𝜖* may, however, be performed to maximize other desirable features. For example, in order reduce the number segments corrupted by noise considered for analysis, one may want to use a lower *𝜖*. This comes to the cost of fewer segments accepted for analysis and of more excluded segments with nystagmus oscillations.

The waveform reconstruction of the data presented in Fig. 4, and illustrated in Fig. 9, captures a representative waveform. By using only three harmonics (five features) it is possible to capture the main properties of the waveform. In this case, such properties include the presence of a foveation period, different frequency for the various waveforms, and the direction of the fast phase. The reconstruction method used in this work is based on the assumption that there is only one type of waveform (according to the classification by Dell’Osso and Daroff (1975)) recorded at each fixation target, which is not necessarily true. In order to use this method for reconstruction of recordings with multiple waveforms, an alternative waveform reconstruction approach would be needed.

The examples representing the estimated *R*_1_ and $\delta _{\phi ^{\prime \prime 2}}$ parameters as polar coordinates, Figs. 7 and 8, illustrate that *𝜖* can be used to separate oscillations from disturbances. There are a few things to take into consideration when evaluating the performance of the presented method on real data. First, it is not possible to know what the true parameter values should be. In order to perform an evaluation of the method on real signals, some assumptions are required. In this case, the assumption is that for a recording of a specific fixation target, the model parameters should be reasonably close to each other for the accepted segments, and they should be separated for the rejected segments. Second, the rejection of segments should be justified. For example, if only one segment is accepted, and all other segments are rejected, then the spread of the parameter values for the accepted segments would be zero. However, this would likely not be useful, since there would be no data left to analyze. As is illustrated in Fig. 7, the parameter values of the accepted segments (blue circles) are more concentrated to a certain position inside the unit circle compared to the parameter values of the rejected segments (red squares). As illustrated in Fig. 10, the rejection of segments is focused on finding segments in the signal where the waveform morphology is difficult to define, e.g., due to a change in waveform morphology. Although there are cases of nystagmus where the waveform changes, e.g., periodic alternating nystagmus (PAN), the time it takes for a waveform to change in PAN is usually several minutes (Leigh & Zee, 2015). The ability to detect waveform changes is determined by the segment length, *N*_*b*_, which in this work equals 0.67 s, and the *𝜖*-value. As can be observed in Fig. 10b, there are multiple waveforms embedded in the signal, and the model is able to capture some of the changing waveforms, although some are rejected. If a shorter segment length is chosen, a better waveform change resolution is obtained, however, this will lead to a decrease in frequency estimation performance. If the *𝜖* is increased, waveform changes will be easier to detect, but at the expense of a higher inclusion rate of non-nystagmus segments. Depending on the desired application of the method proposed in this work, the values of *N*_*b*_ and *𝜖* could vary from the suggestions made in this work.

**Table 3:** Parametrization results from the reconstruction of the waveforms from Dell’Osso and Daroff (1975). The phases are presented in radians

Waveform	*a*_1_	*a*_2_	*a*_3_	*a*_4_	*a*_5_	*a*_6_	*ϕ*_1_	*ϕ*_2_	*ϕ*_3_	*ϕ*_4_	*ϕ*_5_	*ϕ*_6_
Asymmetric pendular (AP)	0.93	0.26	0.07	0.02	0.01	0.	1.13	2.22	3.2	4.12	− 1.17	− 1.16
Bidirectional jerk - left (BDJ-L)	0.6	0.16	0.16	0.07	0.07	0.05	1.29	3.59	− 0.56	2.85	− 0.69	2.93
Bidirectional jerk - right (BDJ-R)	0.6	0.16	0.16	0.07	0.07	0.05	1.85	0.45	2.58	− 0.29	2.45	− 0.21
DJ-L	0.63	0.32	0.21	0.28	0.12	0.09	3.04	2.97	2.87	2.59	2.71	2.64
Dual jerk - right (DJ-R)	0.63	0.32	0.21	0.28	0.12	0.09	− 0.1	− 0.17	− 0.27	− 0.55	− 0.44	− 0.5
Extended foveation - left (EF-L)	0.64	0.28	0.21	0.16	0.12	0.1	2.49	2.69	2.69	2.58	2.52	2.5
EF-R	0.64	0.28	0.21	0.16	0.12	0.1	− 0.65	− 0.45	− 0.45	− 0.57	− 0.62	− 0.64
Jerk - left (J-L)	0.67	0.33	0.21	0.16	0.12	0.1	2.98	2.83	2.71	2.55	2.39	2.27
Jerk - right (J-R)	0.67	0.33	0.21	0.16	0.12	0.1	− 0.17	− 0.32	− 0.43	− 0.59	− 0.75	− 0.87
Pendular (P)	1.02	0.02	0.01	0.03	0.02	0.01	1.63	3.32	− 0.94	1.09	4.14	4.61
Pendular with foveating saccades (P_*FS*_)	0.82	0.21	0.1	0.07	0.04	0.03	1.62	0.76	− 1.32	2.94	1.11	4.61
Pseudo cycloid - left (PC-L)	0.64	0.29	0.16	0.1	0.08	0.06	1.87	− 0.54	3.27	0.63	4.1	1.29
Pseudo cycloid - right (PC-R)	0.64	0.29	0.16	0.1	0.08	0.06	− 1.27	2.6	0.13	3.77	0.96	4.43
Pseudo jerk - left (PJ-L)	0.75	0.27	0.1	0.06	0.05	0.04	2.19	1.08	− 0.36	3.77	2.06	0.21
Pseudo jerk - right (PJ-R)	0.75	0.27	0.1	0.06	0.05	0.04	− 0.95	4.22	2.78	0.63	− 1.08	3.35
Pseudo pendular (PP)	0.88	0.07	0.14	0.05	0.05	0.03	1.02	− 0.84	2.0	0.02	2.69	0.56
PP_*FS*_	0.84	0.19	0.11	0.09	0.02	0.04	1.21	− 0.19	2.88	1.22	− 1.23	2.1
T	0.73	0.01	0.1	0.0	0.05	0.0	1.75	− 0.81	2.13	3.96	2.27	4.33

There are some limitations in terms of the simulated signals generated for the ED. The reconstruction of these waveforms from the original work (Dell’Osso & Daroff, 1975) introduced some artifacts, especially ringing. This is observed for the EF-R waveform in Fig. 2. While this may look like a deviation from the original illustration, these waveforms still serve their purpose for modeling. One of the important characteristics of the nystagmus cycle is whether a foveation period exists and the proportion of this period relative to the cycle duration. These characteristics are well captured by the model presented in this work.

Overall, this method can be used to model and detect changes in waveform morphology, which may be useful information when evaluating different treatment strategies for nystagmus patients, as well as for comparison of eye-movement patterns between different patients.

## Conclusions

The normalized segment error (NSE) and the normalized waveform model (NWM) have been shown to capture the most important features of the nystagmus oscillations. The NSE produced good results for the simulated signals, and the NWM is useful for describing real nystagmus recordings. The method presented in this work may be used to model entire nystagmus signals to be used as a preprocessing step for other models or as a tool to classify different nystagmus waveforms.

## Appendix

### Template waveform reconstruction

1. The greyscale image is thresholded such that all pixels with a greyscale value smaller than 100 is set to 0 (black) and all other pixels are set to 255 (white).
2. The one-dimensional signal is created by running through the columns of the signal and assigning the amplitude of each row the corresponding column value.
3. A four-point median filter is applied in order to remove any outliers from the reconstruction.
4. For each waveform, the start and stop of one cycle is determined by manual annotation by one of the authors [WR].
5. The mean is subtracted from the extracted waveform and subsequently divided by the maximum of the absolute value.
6. The signal is then resampled to create a 10-s-long 5-Hz signal using a sampling frequency of 1000 Hz.
7. Finally, the signals from step 6 were parametrized from 15 harmonics using Fourier analysis.
